# A Hybrid Model for Temperature Prediction in a Sheep House

**DOI:** 10.3390/ani12202806

**Published:** 2022-10-17

**Authors:** Dachun Feng, Bing Zhou, Shahbaz Gul Hassan, Longqin Xu, Tonglai Liu, Liang Cao, Shuangyin Liu, Jianjun Guo

**Affiliations:** 1 Guangzhou Key Laboratory of Agricultural Products Quality & Safety Traceability Information Technology, Zhongkai University of Agriculture and Engineering, Guangzhou 510225, China; 2 College of Information Science and Technology, Zhongkai University of Agriculture and Engineering, Guangzhou 510225, China; 3 Academy of Intelligent Agricultural Engineering Innovations, Zhongkai University of Agriculture and Engineering, Guangzhou 510225, China

**Keywords:** intensive culture, temperature prediction, XGBoost algorithm, particle swarm optimization, principal component analysis

## Abstract

**Simple Summary:**

In intensive sheep farming, the temperature is an important indicator of the healthy growth of sheep. The key to ensuring the healthy growth of sheep in a stress-free environment is to grasp the changing trend in the sheep-house temperature in time and adjust in advance. In order to solve this problem, we use machine learning technology to establish a combined prediction model to accurately predict the temperature of the sheep barn. The result shows that the combined prediction model has good stability and high accuracy. Additionally, it can be extended to the prediction research of other environmental parameters of other animal houses, such as pig houses and cow houses, in the future.

**Abstract:**

Too high or too low temperature in the sheep house will directly threaten the healthy growth of sheep. Prediction and early warning of temperature changes is an important measure to ensure the healthy growth of sheep. Aiming at the randomness and empirical problem of parameter selection of the traditional single Extreme Gradient Boosting (XGBoost) model, this paper proposes an optimization method based on Principal Component Analysis (PCA) and Particle Swarm Optimization (PSO). Then, using the proposed PCA-PSO-XGBoost to predict the temperature in the sheep house. First, PCA is used to screen the key influencing factors of the sheep house temperature. The dimension of the input vector of the model is reduced; PSO-XGBoost is used to build a temperature prediction model, and the PSO optimization algorithm selects the main hyperparameters of XGBoost. We carried out a global search and determined the optimal hyperparameters of the XGBoost model through iterative calculation. Using the data of the Xinjiang Manas intensive sheep breeding base to conduct a simulation experiment, the results show that it is different from the existing ones. Compared with the temperature prediction model, the evaluation indicators of the PCA-PSO-XGBoost model proposed in this paper are root mean square error (RMSE), mean square error (MSE), coefficient of determination (*R*^2^), mean absolute error (MAE) , which are 0.0433, 0.0019, 0.9995, 0.0065, respectively. RMSE, MSE, and MAE are improved by 68, 90, and 94% compared with the traditional XGBoost model. The experimental results show that the model established in this paper has higher accuracy and better stability, can effectively provide guiding suggestions for monitoring and regulating temperature changes in intensive housing and can be extended to the prediction research of other environmental parameters of other animal houses such as pig houses and cow houses in the future.

## 1. Introduction

In Xinjiang, sheep meat production will reach 603,200 tons by 2020 [1]. The Xinjiang sheep business has been essential in alleviating poverty and sustaining and stabilizing houses’ income development [2]. The expansion of science and technology, as well as the development of novel breeding concepts, have altered the sheep breeding paradigm from traditional single-family free-range breeding to large-scale and intense breeding [3]. Temperature is a critical environmental indicator in intensive housing. In winter, the minimum temperature in the sheep house should be above 10 °C, and in summer, the temperature should not exceed 30 °C. If the temperature is too high, a sheep’s heat dissipation is impaired, affecting feed intake and utilization. Bacteria breed and cause ectoparasitic and respiratory system diseases in sheep; if the temperature is too low, it is detrimental to a lamb’s health and survival. The proportion of feed consumed to maintain body temperature increases, negatively affecting sheep production and reproductive performance [4]. However, the temperature in the sheep house is time-varying, lags, exhibits significant interference, and exhibits multiple coupling [5]. The frequently employed linearization model cannot accurately predict the dynamic change law of the sheep house environment. The model’s poor accuracy directly influences the sheep house’s environmental management performance [6]. As a result of this complex problem, it is critical to use intelligent algorithms to predict the sheep house’s temperature accurately, understand the trend of temperature changes over time, and plan preventive and control measures to ensure the flock of sheep grows in a healthy environment.

Many scholars have explored the temperature prediction model in recent years and achieved remarkable results in pig houses, greenhouses, room temperature, and water temperature [7,8,9,10,11,12,13,14,15,16,17,18,19,20,21]. Xie Q et al. developed a new dynamic heat exchange model using EBE to accurately predict temperature changes in pig houses [7]. Ortega verified the indoor temperature predictions based on the ARIMA model under outdoor temperature changes. The indoor temperature prediction of the animal area in a conventional weaning piglet house is robust [8]. Fang Z et al. designed an LSTM-based Seq2Seq model to achieve multi-step advanced prediction of indoor temperature [9]. Elmaz F et al. proposed that a CNN-LSTM architecture was used to predict indoor temperature, and the proposed architecture showed excellent stability [10]. Zhou designed a novel convex bidirectional extreme learning machine (CB-ELM) model for greenhouse temperature and humidity prediction, which was proven experimentally to be a viable and effective approach for determining the temperature and humidity of solar greenhouses [11]. Moon T et al. used transfer learning to adjust the previously trained BiLSTM microclimate prediction model for greenhouses [12]. Shamkhani H et al. modeled air temperature through a generalized regression neural network. According to the findings, the GRNN model is a reliable tool for temperature prediction [13]. Jung D H et al. applied the RNN-LSTM model more accurately [14]. Cai W et al. proposed a model based on Light GBM to simulate the internal temperature of the greenhouse [15]. Li designed a time-delay neural network and Ellman neural network (ENN) based indoor temperature prediction system [16]. M Martnez-Comesaa et al. used the multi-objective genetic algorithm NSGA-II to optimize the MLP neural network. They obtained its most precise temperature prediction regarding error and complexity [17]. Li et al. used the Elman neural network, a multi-step prediction model for prediction in a closed environment [18]. Xu L et al. applied the IABC-WTMM model to predict water temperature in shrimp culture, overcoming traditional WTMM blindness and limitations of model parameter selection [19]. Renata Graf et al. developed an integrated model for temperature prediction in rivers by coupling WT and ANN. This model’s excellent performance is especially evident under extreme conditions [20]. Alhamid, M.I. et al. correctly predicted the hot water temperature at the generator’s input using a feedforward backpropagation neural network. The correlation between the anticipated and experimental values was satisfactory [21]. Even though the temperature prediction models proposed by these scholars have good accuracy, there are still issues with their real application, such as sluggish model training, the optimization algorithm is easy to fall into the local optimum when searching for the global optimum solution (That is, it seems to be the optimal solution at present, but it is not the global optimal solution), and the algorithm’s high computing cost.

Compared to typical machine learning algorithms, the XGBoost technique uses the CPU’s multi-threading capability. It incorporates a regularization term to regulate the algorithm’s complexity and prevent the model from overfitting. The benefits are substantial and may significantly increase the model’s accuracy. XGBoost is a gradient descent algorithm that combines multiple weak classifiers into a single strong classifier. As a result of enhancing learning capacity, XGBoost is extensively employed in prediction research across various domains [22,23,24,25]. Using SVMD and XGBoost, Wang Y. et al. increased the accuracy of short-term load prediction by developing a short-term load forecasting model for industrial clients [23]. Liu, Y. et al. proposed a prediction model of odor concentration in chicken houses based on XGBoost, and verified the effectiveness of the algorithm [24]; Obsie E.Y. et al. built a wild blueberry yield prediction method based on XGBoost, which greatly improved the prediction accuracy of blueberry yield [25]. As a result, the XGBoost algorithm was chosen for this experiment to investigate temperature prediction in the sheep house to maximize its benefits in complicated prediction issues and improve temperature forecast accuracy.

The hyperparameters in the XGBoost model directly impact the model’s prediction performance. The performance of models trained with various values is often considerably different, making it critical to choose optimal hyperparameters for the XGBoost model [26]. The benefits of the particle swarm optimization technique are its rapid search speed, high efficiency, resilience, simplicity of the algorithm, and ease of convergence. It is extensively employed in the domains of energy and agriculture [27,28,29]. Zhang, Y. et al. presented the particle swarm optimization technique to improve the parameters of back propagation neural network (BPNN) to forecast daily global solar radiation, solving the difficulties of parameter uncertainty, gradient descent, local optimization, etc. [27]; Huang, Y. et al. proposed particle swarm optimization to optimize support vector regression (SVR), which can accurately capture power peak loads [28]; Zhu, B. et al. optimized the parameters of extreme learning machine (ELM) through particle swarm algorithm, which improved the accuracy and running speed of the crop evapotranspiration prediction model [29].

Due to the sheep house breeding environment is complicated, and some environmental elements interact to impact temperature, some redundancy or information overlaps across environmental parameters. Suppose all components are included directly in the model. In that case, this may result in duplicate data and an overly complex model building. Simultaneously, it succumbed to model overfitting, resulting in suboptimal temperature prediction for sheep breeding environment parameters. As a result, the PCA dimension reduction technique filters the original environmental elements, eliminates their association, and prevents the dimension catastrophe produced by too high feature dimensions, which is critical for improving the model’s accuracy.

As a consequence of prior research, this work employs PCA, PSO, and Extreme Gradient Boosting to overcome the experience and unpredictability associated with XGBoost model hyperparameter selection. The temperature prediction of Xinjiang sheep houses using PCA-PSO-XGBoost is suggested in combination with XGBoost (Extreme Gradient Boosting, XGBoost). PCA removes large temperature-influencing components, and PSO is applied to do a global search for XGBoost hyperparameters. The optimized model is applied to the Xinjiang Manas intensive sheep breeding base. The prediction results are compared to those obtained using the conventional BPNN, SVR, XGBoost, PSO-BPNN, PSO-SVR, and PSO-XGBoost models, demonstrating that the model can effectively provide technical support for temperature change monitoring and regulation in intensive sheep breeding.

## 2. Materials and Methods

### 2.1. Experimental Area

**Figure 1:**
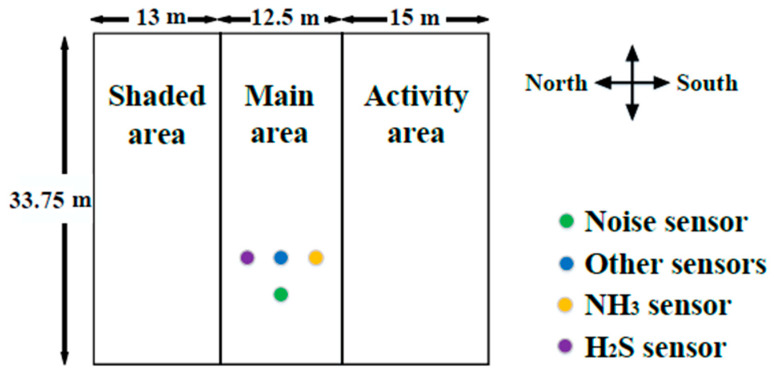
Schematic diagram of the Xinjiang sheep house monitoring.

In our study, the breeding group ewe barn No. 5 in the Xinjiang Uygur Autonomous Region Manas Xin’ao Animal Husbandry Co., Ltd. base (44°27′18″ north latitude, 86°10′47″ east longitude, Changji Hui Autonomous Prefecture, China) was used as the experimental data area. A comprehensive and intensive breeding production base for Suffolk sheep integrates breeding, production, and sales. This study selects a semi-enclosed Suffolk sheep house with an area of about 422 m^2^. The four walls of the house are made of a brick–concrete structure, mainly made of bricks, steel bars, and concrete. The top surface is a steel plate structure, which is mainly composed of steel beams, steel columns, steel trusses, and other components made of section steel and steel plates, and the ground is a soil structure. The sheep house is divided into three areas: the main area, a shaded area, and an activity area. The sheep are concentrated in the main area for closed breeding in winter. This is to prevent the cold wind from entering the sheep house in winter, thereby reducing the heat loss of the sheep, preventing the sheep from frostbite or even freezing to death. Sensors such as temperature and humidity, noise, light intensity, PM2.5, PM10, TSP, CO_2_, NH_3_, and H_2_S are installed in the main area to monitor the environmental parameters of the sheep barn online. The ammonia sensor is 3.2 m from the ground, the hydrogen sulfide sensor is 3.7 m from the ground, the noise sensor is 3.1 m from the ground, and other sensors are 3.3 m from the ground as shown in Figure 1.

### 2.2. Experimental Data Preprocessing

When many variables in a sheep house environment have varying magnitudes, it is necessary to normalize and eliminate them from the data set to increase the forecast accuracy of these variables [30]. The normalized calculation formula is as follows:

(1) $$X_{i}{}^{*}=\frac{X_{i}-\bar{X}}{X_{s}}$$

where, $\bar{X}$ is the mean, and $X_{S}$ standard deviation and $X_{i}^{*}$ is ith normalized value.

### 2.3. Construction of a Combined Forecasting Model for Sheep House

#### 2.3.1. XGBoost Algorithm

Friedman introduced the Gradient boosting decision tree technique in 2001 [31]. Gradient descent is used to generate a new structure based on these prior trees to reduce the objective function [32]. Extreme gradient boosting (XGBoost) is an acronym for Extreme Gradient Boosting Decision Tree.

It is an ensemble tree regression and classification model. When XGBoost is applied to a regression problem, it generates new regression trees. The residuals of the previous model are then fitted utilizing the newly generated CART tree. A fully trained model has a total number of trees of K in it. The total estimated value is determined by aggregating the findings for each tree [32].

The objective function of XGBoost consists of a loss function and a regularization term. Use to denote the loss error and denote the regularization term. In order to control the complexity of the tree model and prevent overfitting, Chen and Guestringave carried out the following derivation process for the objective function of XGBoost. The objective function adds a new function $f_{m}$ through continuous iteration so that the final result is minimized. The objective function in the mth iteration can be expressed as:

(2) $$Obj^{\left(m\right)}=\sum_{i=1}^{n}loss\left(y_{i},{\bar{y}}_{i}\right)+\sum_{m=1}^{M}\Phi \left(f_{m}\right)$$

In Formula (2),$\sum_{i=1}^{n}loss\left(y_{i},{\bar{y}}_{i}\right)$ is used to measure the error between the predicted value ${\bar{y}}_{i}$ and the true value $y_{i}$. $\sum_{m=1}^{M}\Phi \left(f_{m}\right)$ is the regularization term, which represents the sum of the complexity of each submodel, and it is used to prevent the model from overfitting

The calculation formula of the regularization term is:

(3) $$\Phi \left(f_{m}\right)=\gamma T+\frac{1}{2}\lambda \sum_{l=1}^{T}\omega_{l}{}^{2}$$

In Formula (3), γ is the regularization parameter of the number of leaf nodes, which is used to control the number of leaf nodes; T is the number of leaf nodes; λ represents the regularization parameter of leaf node weights, which is used to control the weight of leaf nodes to prevent the result from overfitting; $\omega_{l}$ is the weight of the leaf node of each tree.

After *m* iterations, the resulting objective function in Formula (2) can be expressed as:

(4) $$Obj^{\left(m\right)}=\sum_{i=1}^{n}loss\left(y_{i},{\bar{y}}_{i}^{\left(m-1\right)}+f_{m}\left(x_{i}\right)+\Phi \left(f_{m}\right)+Const\right)$$

The objective function obtained by Formula (4) after Taylor series expansion and removal of high-order infinitesimal terms is:

(5) $$Obj^{\left(m\right)}=\sum_{i=1}^{n}\left[loss\left(y_{i},{\bar{y}}_{{}_{i}}^{\left(m-1\right)}\right)+p_{i}f_{m}\left(x_{i}\right)+\frac{1}{2}q_{i}f_{m}^{2}\left(x_{i}\right)\right]+\Phi (f_{m})+Const$$

In Formula (3), $p_{i}=\partial {\bar{y}}^{\left(m-1\right)}loss\left(y_{i},{\bar{y}}_{i}^{\left(m-1\right)}\right)$, $q_{i}=\partial^{2}{\bar{y}}^{\left(m-1\right)}loss\left(y_{i},{\bar{y}}_{{}_{i}}^{\left(m-1\right)}\right)$.

Since the result of the objective function needs to be minimized, the constant term has no influence on the formula. After removing the constant term, the objective function can be simplified as:

(6) $$\begin{array}{l} Obj^{\left(m\right)}=\sum_{i=1}^{n}\left[p_{i}f_{m}\left(x_{i}\right)+\frac{1}{2}q_{i}f_{m}^{2}\left(x_{i}\right)\right]+\Phi (f_{m})\\ =\sum_{i=1}^{n}\left[p_{i}w_{q}\left(x_{i}\right)+\frac{1}{2}q_{i}w_{{}_{q}}^{2}\left(x_{i}\right)\right]+\gamma T+\lambda \frac{1}{2}\sum_{l=1}^{T}w_{l}^{2}\\ =\sum_{i=1}^{T}\left[\left(\sum_{i\in I_{l}}p_{l}\right)w_{l}+\frac{1}{2}\left(\sum_{i\in I_{l}}q_{i}+\lambda \right)w_{l}^{2}\right]+\gamma T \end{array}$$

In Formula (6), let $P_{l}=\sum_{i\in I_{l}}p_{l}$, $Q_{l}=\sum_{i\in I_{l}}q_{l}$, solving Equation (6) to achieve the objective function as:

(7) $$Obj^{\left(m\right)}=\sum_{l=1}^{T}\left[P_{l}w_{l}+\frac{1}{2}\left(Q_{l}+\lambda \right)w_{l}^{2}\right]+\gamma T$$

The value of $w_{l}$ obtained by derivation of the objective function is as follows $w_{l}=-\frac{P_{l}}{Q_{l}+\lambda }$. Replacing it into Equation (7) to solve, and achieve the final objective function as:

(8) $$Obj^{\left(m\right)}=-\frac{1}{2}\sum_{l=1}^{T}\frac{P_{l}^{2}}{Q_{l}+\lambda }+\lambda T$$

#### 2.3.2. Particle Swarm Optimization Algorithm

**Figure 2:**
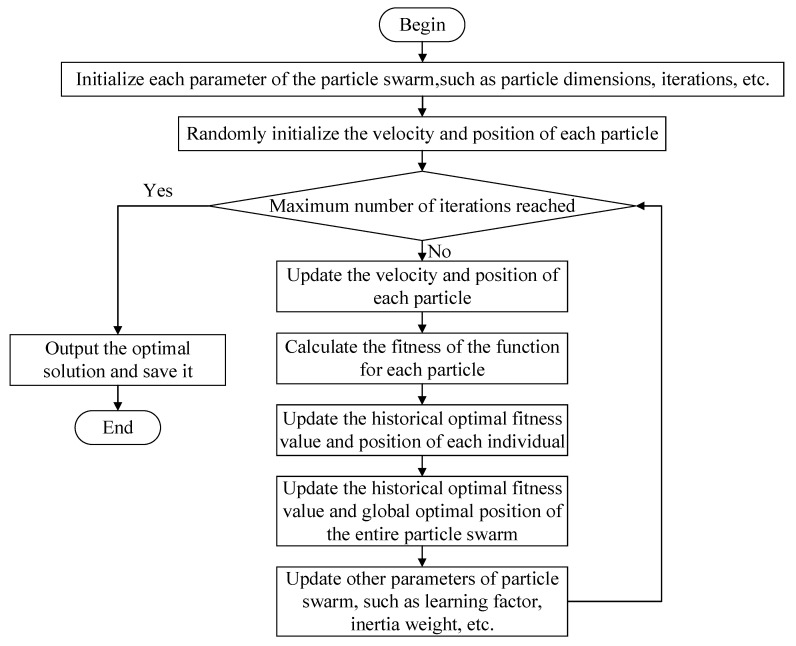
Flow chart of particle swarm optimization algorithm.

A particle swarm optimization algorithm is a swarm intelligence optimization algorithm based on bird social behavior proposed by Eberhart and Kennedy in 1995 [33]. It is currently used in solving various optimization problems with remarkable results [34]. The basic idea is that each bird in a flock is seen as a particle, a candidate solution to the problem to be solved. By continuously adapting to the environment and adjusting the speed and position of each individual, the optimal solution is continuously approached [35] to realize the optimization of the individual in the solution space. Flow of particle swarm optimization algorithm is shown in Figure 2. The particle swarm optimization algorithm mainly consists of the following steps, which loop all the time to satisfy the termination condition:

Step 1: Determine the fitness of each particle.
Step 2: Continuously update the best individual extrema $z_{j}$ and the optimal global solution $z_{g}$
Step 3: $S_{J}^{T}$ indicates the direction and distance for the next iteration of each particle. $Y_{j}^{T}$ represents the current state of each particle. Update $S_{J}^{T}$ and $Y_{j}^{T}$ of each particle.

For the PSO algorithm, it is assumed that there are M particles in the K-dimensional target search space, and these M particles representing candidate solutions form a particle swarm.

The K-dimensional vector $Y_{j}={\left[Y_{j1},Y_{j2},..,Y_{jK}\right]}^{T}$ can be used to represent the information of the jth particle, $S_{j}={\left[S_{j1},S_{j2},...,S_{jK}\right]}^{T}$ is the velocity of these particles. The particles cooperate and share information according to the two extreme values updated in each iteration. The fitness value of each particle is calculated, and the individual extreme value is the optimal fitness value determined by the jth particle in the search space. The global extrema is the best position found by the entire particle swarm among these individual extrema, denoted as: $z_{g}={\left[z_{g1},z_{g2},...,z_{gK}\right]}^{T}$. The particle updates these two extrema according to Equations (9) and (10), to update its velocity $S_{j}^{T}$ and position $Y_{j}^{T}$.

(9) $$S_{j}^{T+1}=\mu S_{j}^{T}+\varepsilon_{1}\left(z_{j}{}^{T}-Y_{j}^{T}\right)+\varepsilon_{2}\left(z_{g}^{T}-Y_{j}^{T}\right)$$

(10) $$Y_{j}^{T+1}=Y_{j}^{T}+Y_{j}^{T+1}$$

In Formulas (9) and (10), μ stands for inertia weight, $\varepsilon_{1}$ and $\varepsilon_{2}$ are determined by $c_{1}r_{1}$ and $c_{2}r_{2}$ respectively, T represents the Tth iteration, $r_{1}$ and $r_{2}$ are random numbers from 0 to 1. In addition, $c_{1}$ represents the individual learning factor and $c_{2}$ stands for the population learning factor.

#### 2.3.3. Model Performance Evaluation

The performance evaluation of the Xinjiang sheep house temperature prediction model based on PCA-PSO-XGBoost uses Formulas (11) to (14) to calculate each model [36]. The expressions of these specific evaluation indicators are as follows:

(11) $$MSE=\frac{1}{b}\sum_{j=1}^{b}{\left(a_{j}-{a^{\prime }}_{j}\right)}^{2}$$

(12) $$RMSE=\sqrt{\frac{1}{b}\sum_{j=1}^{b}{\left(a_{j}-{a^{\prime }}_{j}\right)}^{2}}$$

(13) $$MAE=\frac{1}{b}\sum_{j=1}^{b}\left|a_{j}-{a^{\prime }}_{j}\right|$$

(14) $$R^{2}=\frac{\sum_{j=1}^{b}{\left({a^{\prime }}_{j}-\bar{a}\right)}^{2}}{\sum_{j=1}^{b}{\left(a_{j}-\bar{a}\right)}^{2}}$$

In Formulas (11) to (14), $a_{j}$ is the actual value of the *j*th data, ${a^{\prime }}_{j}$ is the predicted value of the jth data predicted by the model, $\bar{a}$ is the average of these data, and b represents the total of the b data.

It can be observed from the preceding calculation that MSE represents the average of the square of the discrepancy between the estimated value and the actual value. The higher the MSE value, the greater the discrepancy between the predicted value of the model and the actual value. The higher the fitting effect of the model is; the MAE reflects the average value of the absolute error. The bigger the MAE number, the larger the inaccuracy of the model’s anticipated value; *R*^2^ is the baseline for measuring model quality, and its value range is typically [0, 1]. The greater the *R*^2^, the stronger the correlation between the model’s estimated value and the actual value, and the nearer it is to 1, the ideal. *R*^2^ represents how well the model fits the original data; in addition, the value range is generally [0, 1]. The higher the *R*^2^ value, the closer the model calculation result is to 1, and the stronger the correlation between the model-predicted value and the actual value is.

## 3. Result and Analysis

### 3.1. Experimental Environment

**Figure 3:**
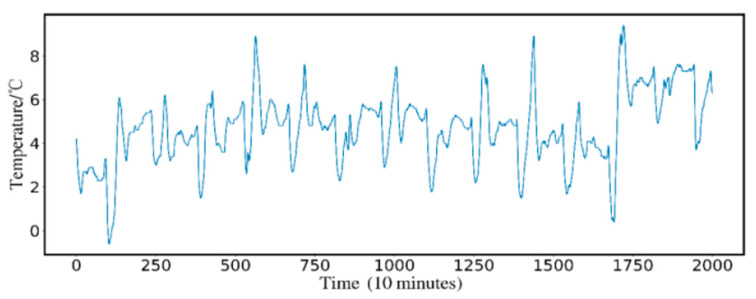
Raw temperature data.

Taking the temperature of the ewe house of the No. 5 breeding group of Xinjiang Manas Xin’ao Animal Husbandry Co., Ltd. as the research object, using the Internet of Things cloud service platform for environmental monitoring of sheep house breeding developed by Zhongkai Agricultural Engineering College, the data is collected with an interval of 10 min. Two thousand sheep barn parameter data from 8 February 2021 to 22 February 2021 were used as the experimental data, including air temperature, air humidity, carbon dioxide concentration, PM2.5 index, PM10 index, light intensity, noise, TSP index, for parameters such as hydrogen sulfide concentration, in which 1400 prior time periods were the training set. The last 600 time periods were set to realize the temperature prediction in the sheep house for the next 10 min. The raw temperature data are shown in Figure 3. The ordinate corresponds to the temperature of the sheep house, and the abscissa is in units of 10 min, corresponding to 2000 time periods during the period from 17:11 on 8 February to 17:32 on 22 February:

The experimental setup includes Intel(R) Core (TM) i7-6400U processor (Santa Clara, CA, USA), 2.30GHz CPU frequency, 16 GB memory, Windows10 (64-bit) (Microsoft, Redmond, WA, USA) operating system, the integrated development environment is Anaconda3, the programming language is python3.6 (64-bit), and the experiment uses the Keras, Sklearn, and XGBoost packages to realize the temperature prediction of Xinjiang sheep house based on the PCA-PSO-XGBoost model. Through repeated tests, the PSO parameters were set as follows: The population size N is set at 20, the maximum number of iterations T is set at 20, the local search factor C1 is set at 2, and the global search factor C2 is set at 2, and the self-weight factor w = 0.4; the learning rate of the XGBoost model and the maximum depth of the tree is globally searched by the PSO optimization algorithm. The maximum depth max_depth = 9, the maximum number of trees n_estimators is 1000, and other parameters are set to default values.

### 3.2. Experiment Procedure

**Figure 4:**
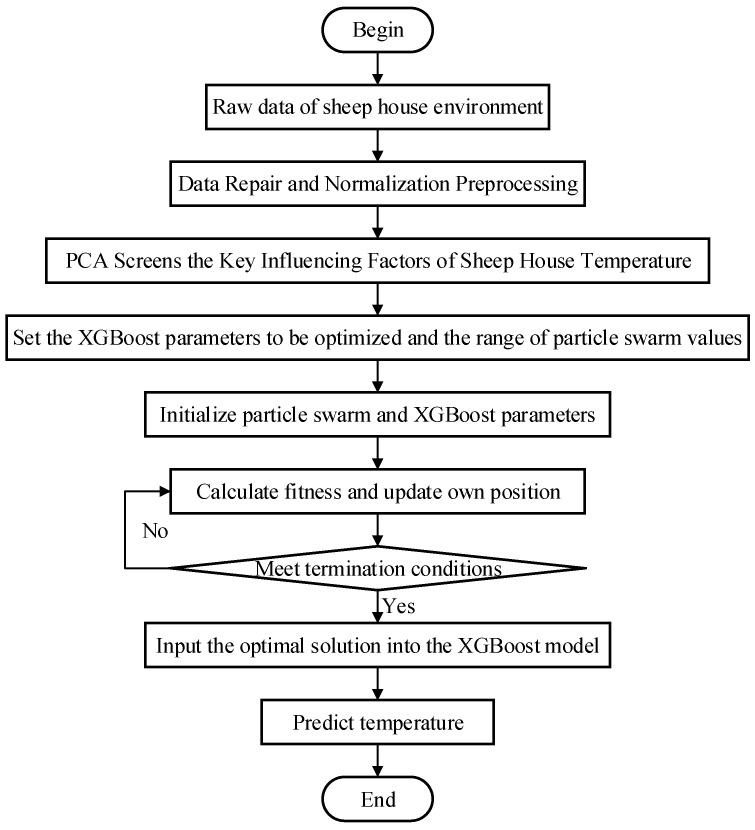
Temperature prediction flow chart based on PCA-PSO-XGBoost.

The experiment organically combined the PCA dimension reduction algorithm, PSO optimization algorithm, and the XGBoost model to build a temperature prediction model for the Xinjiang sheep house based on PCA-PSO-XGBoost to increase temperature prediction accuracy. Standardized processing, using principal component analysis to screen the key influencing factors of sheep house temperature in Xinjiang, eliminating redundant information among environmental factors, and simplifying the structure of the XGBoost model. Once the PSO optimization method has been used to optimize the XGBoost model’s learning rate, tree depth, and other parameters to enhance accuracy, the best solution is then replaced with the XGBoost model to produce the prediction outcome. The specific steps are shown in Figure 4:

Step 1: Standardize the temperature time series data for the Xinjiang sheep houses and split them into test and training sets by time. The first 70% of these 2000 time periods is the training set, while the last 30% is the test set.
Step 2: Use principal component analysis to screen the key influencing factors of sheep house temperature in Xinjiang, eliminate redundant information among environmental factors, and simplify the model structure.
Step 3: Initialize the particle swarm and XGBoost model parameter ranges.
Step 4: Based on the training set, PSO optimization was used to optimize the optimal learning rate of the XGBoost and obtain a temperature prediction model based on PSO-XGBoost. Among them, the preset XGBoost model learning rate parameter range is [0,1]. PSO has 10 rounds of optimization, with 10 particles in each round, and *R*^2^ is used as the fitness.
Step 5: Apply the PCA-PSO-XGBoost model to the Xinjiang sheep house environmental prediction field to monitor and regulate temperature changes in intensive housing.

### 3.3. Result and Discussions

The temperature of the ewe house in Xinjiang Manas Xin’ao Animal Husbandry Co., Ltd.’s No. 5 breeding group is used as the research object. The data were collected using the Internet of Things cloud service platform for monitoring the breeding environment of animals developed by Zhongkai Agricultural Engineering College. Between 8 February and 22 February 2021, 2000 time periods of data were gathered every 10 min, including air temperature, air humidity, carbon dioxide concentration, the PM2.5 and PM10 indexes, light intensity, noise, TSP index, and hydrogen sulfide. These factors forecast the sheep house’s temperature for the following ten minutes. Table 1 contains the raw data of the barn’s breeding environment.

The process is complex in the sheep house breeding environment, where various environmental elements interact to influence the temperature. If all the factors are directly input into the model, the model may be too complicated to construct. The over-fitting problem leads to the low-temperature prediction accuracy of sheep breeding environment parameters. PCA and SPSS statistical analysis tools were used to reduce duplicate information and data correlation. The key influencing factors were extracted by analyzing the sheep house data and reducing the dimension to improve the model’s prediction accuracy effectively. Table 2 shows the eigenvalues of each principal component, variance contribution rate, and cumulative total variance obtained after dimensionality reduction analysis. The principal component analysis is based on selecting components with “eigenvalues” larger than one. As seen in Table 2, The eigenvalues of all three components are larger than one, consistent with the notion of main component extraction. Therefore, the first three components of the original characteristics are identified as replacements for the original variables.

Table 3 presents the factor loading values of every environmental factor for the various main components after rotation using the Kaiser normalized orthogonal rotation technique. The contribution to the first principal component is Total Suspended Particulates (TSP), PM10, PM2.5, and the carbon dioxide concentration and air humidity contribute more to the second principal component.

The air temperature contributes more to the third principal component. Table 4 displays the association between the temperature and other environmental characteristics. Air temperature is inversely proportional to air humidity and positively proportional to carbon dioxide concentration, air humidity, total suspended particle matter (TSP), PM10, and PM2.5. The link between carbon dioxide concentration and air humidity was statistically significant. A significant positive association existed between TSP, PM10, and PM2.5. Therefore, the key influencing factors screened out are air temperature, carbon dioxide concentration, PM10, PM2.5, air humidity, and the total suspended particulate matter (TSP), which are the primary influencing variables of sheep housing temperature, as determined by professionals in sheep breeding.

**Figure 5:**
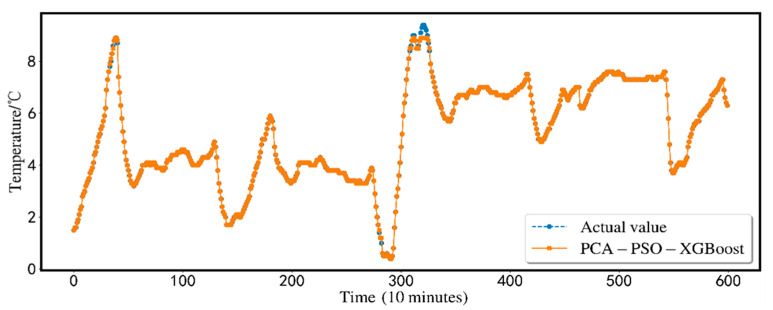
PCA-PSO-XGBoost prediction results.

**Figure 6:**
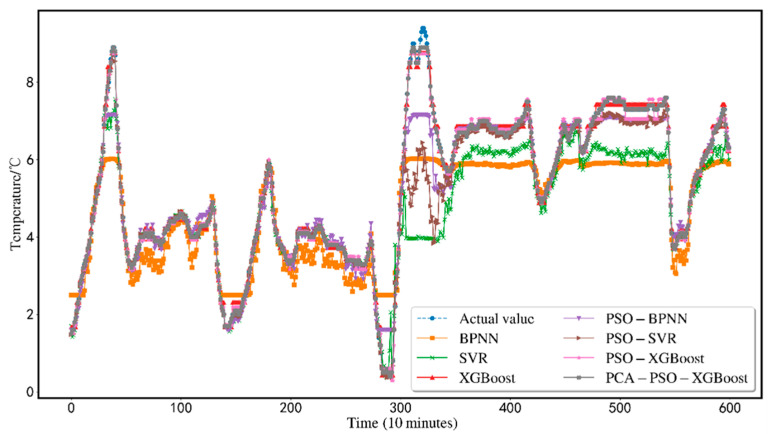
Comparison of prediction results.

The predicted results are shown in Figure 5. In order to show the effectiveness of the proposed scheme, this research conducts a comparative analysis of seven alternative prediction models using the same dataset. Traditional BP-NN, XGBoost, SVR, BP-NN, SVR, and XGBoost based on PSO particle swarm optimization are compared with the XGBoost model (PCA-PSO-XGBoost) based on the PCA for dimensionality reduction and the particle swarm. The comparison is shown in Figure 6. Comparing the three indicators, MSE, MAE, and *R*^2^, the findings of the PCA-PSO-XGBoost prediction model best match the real data. The total error is minimal, the prediction accuracy is high, and great results may be produced.

**Figure 7:**
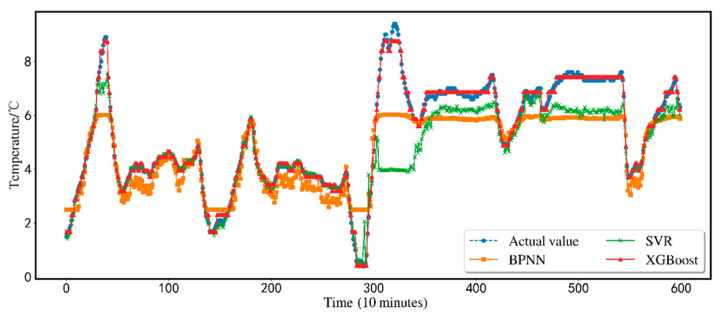
Comparison of SVR, BPNN and XGBoost.

Figure 7. compares a single SVR, a single BPNN, and a single XGBoost model. While SVR and BPNN models are typically reliable, the results of an XGBoost model are still more precise than SVR and BPNN models. The first half of the BPNN prediction curve floats up and down the actual value curve. The fluctuation range is large, but there is an obvious error in predicting the peak value. However, the SVR prediction curve can match the original value curve in the first half, and the prediction effect in the second half is not good. The error with the real value is large.

Although BPNN has a strong nonlinear fitting ability, it has certain advantages for processing unstable data. It can describe small changes in input variables. However, BPNN has poor generalization ability, is very sensitive to initial weights, and is easy to converge to the local minimum, often stagnates in the flat region of the error gradient surface, and the convergence is slow or even unable to converge; SVR is a stable classifier and is easily affected by noise data during training. However, the air temperature in the sheep barn depends on air humidity, carbon dioxide concentration, PM2.5 index, PM10 index, light intensity, noise, TSP, hydrogen sulfide concentration, etc. Environmental factors, which make it sensitive to environmental factors, cause the SVR model to have certain limitations in predicting sheep barn temperature. Additionally, when there are several input variables, it is hard to precisely characterize and manage the influence of minor changes in input parameters on the output results due to the SVR model’s absence of a processing mechanism for missing data. This flaw significantly impacts the accuracy of temperature prediction.

In XGBoost, there is a mechanism for capturing and processing missing values within the algorithm. Different weak learners can be used to process missing values. In addition, XGBoost uses first-order and second-order gradient optimization and introduces L1 and L2 regular terms to control the complexity and reduce over-fitting. XGBoost draws on RF column sampling and introduces a random mechanism to reduce the over-fitting and calculation amount. To sum up, the XGBoost algorithm has feasibility and good fitting ability in the temperature prediction of a sheep house in Xinjiang.

**Figure 8:**
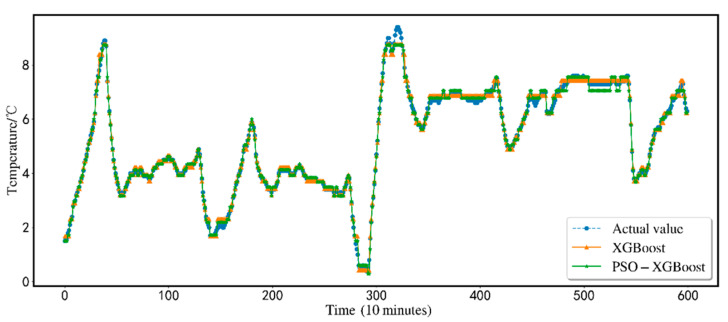
Comparison of XGBoost and PSO-XGBoost.

Figure 8 compares the single XGBoost model with the PSO-optimized XGBoost model. The single XGBoost model does not match the original value curve and the PSO-optimized XGBoost model. In the time period of 350–550, a large horizontal line appears in the prediction result of a single XGBoost model, while PSO-XGBoost can match the true value curve, which shows that the PSO method optimizes the selection of parameters required by the XGBoost model and can improve the model search speed, at the same time it can overcome the blindness and limitations of the traditional XGBoost model in parameter selection, thereby improving the model prediction accuracy.

**Figure 9:**
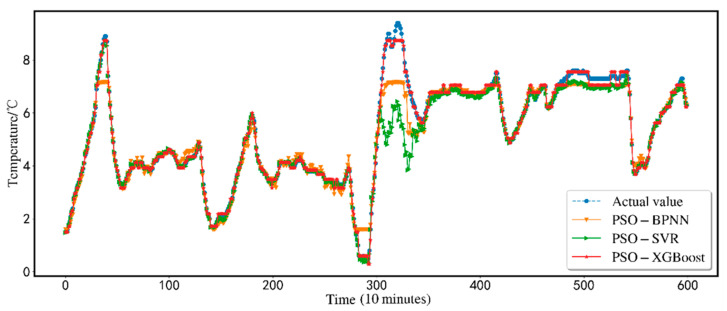
Comparison of PSO-BPNN, PSO-SVR, and PSO-XGBoost.

Figure 9 shows the comparison results of the PSO-SVR, PSO-BPNN, and PSO-XGBoost models. It can be seen intuitively from the figure that the prediction results of the PSO-XGBoost model fit the original value curve. Although the prediction curves of PSO-BPNN and PSO-SVR have good prediction results in the first half, there are small fluctuations in different degrees in the second half. Both BPNN and SVR need to configure multiple parameter items and train more times to achieve better prediction results. It is simple to enter the local optimum when utilizing the PSO method for optimization. The flaws in the algorithm itself cannot be fixed by it efficiently. The XGBoost model has relatively simple parameter settings, strong robustness, good fitting effect, and high model accuracy. As a result, under identical circumstances, the PSO-XGBoost model outperforms the PSO-BPNN and PSO-SVR models.

**Figure 10:**
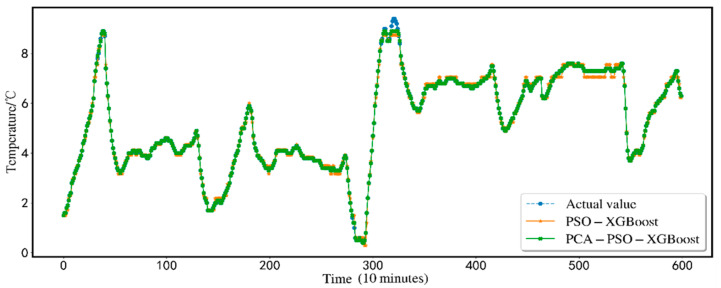
Comparison of PSO-XGBoost and PCA-PSO-XGBoost.

Figure 10 is a comparison diagram of the PSO-XGBoost and the PSO-XGBoost models after PCA dimensionality reduction. As the picture shows, the PSO-XGBoost model’s small fluctuation in the time period of 500–550, the PSO-XGBoost model after PCA dimensionality reduction is more consistent with the original value curve. This is because the environmental index data of the sheep house have been processed by PCA dimensionality reduction. The environmental parameters that have the greatest impact on temperature are screened out. The noise data that interferes with the prediction performance are eliminated. As a result, the distribution of the input prediction model’s primary determinants is more concentrated, leading to maximum prediction accuracy. The PCA-PSO-XGBoost model presented in this study provides the best prediction effect. Simultaneously, it can better fit the nonlinear relationship between the ecological environment influencing factors and temperature of sheep house breeding, which has important guiding significance for sheep breeding.

**Figure 11:**
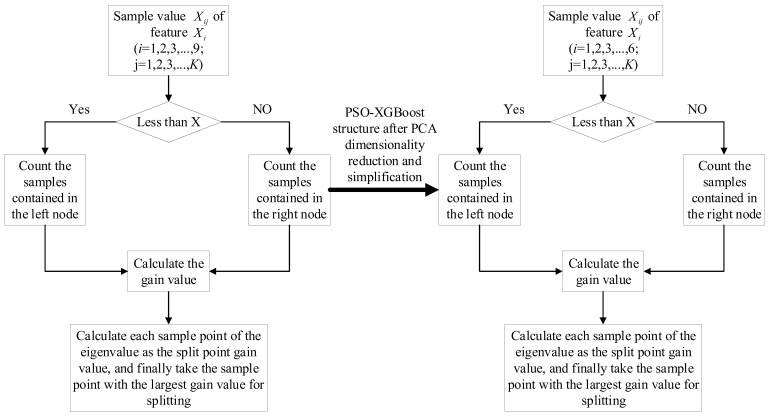
PSO-XGBoost structure after PCA dimensionality reduction and simplification.

Figure 11 shows the PSO-XGBoost structure after PCA dimensionality reduction and simplification. This is reflected in the reduction in the number of variables in the input model. By reducing the nine variables of the original input model into six variables, the computational overhead of the algorithm is reduced, and the prediction accuracy of the model is effectively improved.

Table 5 shows that the PCA-PSO-XGBoost prediction model suggested in this article outperforms other prediction models in terms of each index. A single XGBoost model outperforms a single BPNN and a single SVR model in terms of performance. RMSE, MSE, *R*^2^, and MAE are 89, 99, 70, and 89% higher than those of a single BPNN and 88, 99, and 54% higher than a single SVR, 82%. It shows that under the condition of small samples, XGBoost can better dig out the law of nonlinear temperature change in sheep house environments than BPNN and SVR. The prediction accuracy RMSE, MSE, *R*^2^, and MAE of PSO-XGBoost are 0.1256, 0.0158, 0.9957, and 0.0898, respectively. Compared with PSO-BPNN, RMSE, MSE, *R*^2^, and MAE are improved by 72%, 92%, 5%, and 62%, respectively. The PSO-SVR is improved by 82%, 97%, 15%, and 63%, respectively. Compared with a single XGBoost, the prediction accuracy RMSE, MSE, and MAE of PSO-XGBoost are improved by 8%, 16%, and 13%, respectively. Compared with a single BPNN, the prediction accuracy RMSE, MSE, *R*^2^, and MAE of PSO-BPNN are improved by 64%, 87%, 62%, and 15%, respectively. Prediction accuracy RMSE, MSE, R2, and MAE of the PSO-optimized SVR model increased by 38, 62, 34, and 57% compared to the single SVR model. This demonstrates how a single XGBoost, BPNN, and SVR may significantly increase their prediction accuracy by the PSO optimization method. Compared to the PSO-XGBoost model, the model outputs with PCA dimensionality reduction are more precise. The RMSE, MSE, *R*^2^, and MAE values for prediction accuracy are 0.0433, 0.0019, 0.9995, and 0.0065, respectively. RMSE, MSE, and MAE are higher than those of PSO. XGBoost increases by 66, 88, and 93%, which shows that using the PCA dimensionality reduction algorithm to screen the key environmental factors affecting sheep house temperature can remove the correlation between environmental factors and simplify the model structure, and effectively improve the model accuracy. The findings demonstrate that the PCA-PSO-XGBoost model in this paper has greater accuracy and a better learning effect, can fully mine the hidden information within the data, and can more precisely predict how the temperature will change in the sheep house for the following ten minutes. It offers a trustworthy foundation for deciding temperature prediction and early warning in intensive sheep farming.

In order to illustrate the applicability of the PCA-PSO-XGBoost model at higher temperatures, this paper generalizes the validation on a dataset with a maximum temperature of 22.4 degrees Celsius.

**Figure 12:**
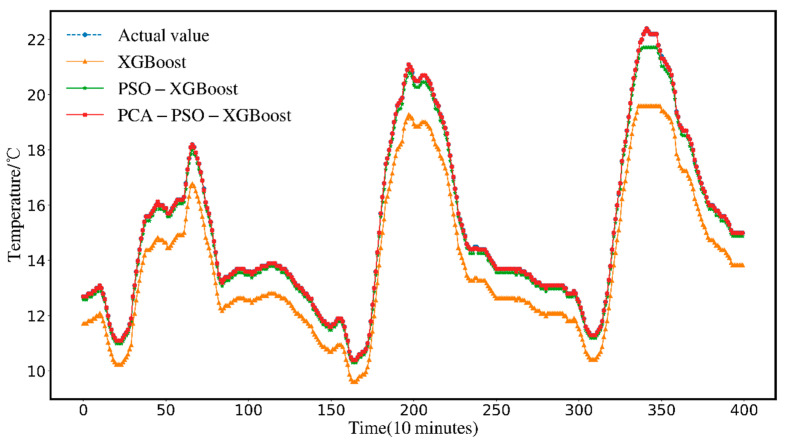
Comparison of prediction results in high temperature dataset.

Figure 12 shows the comparison results of the original data curves with the prediction curves of the single XGBoost model, the PSO-XGBoost model, and the PCA-PSO-XGBoost model. As shown in the figure, the PSO-XGBoost model after PCA dimensionality reduction is more in line with the original value curve and can also fit the original data in the prediction of the temperature peaks of the 60th, 200th, and 350th time segments. As shown in the figure, the PSO-XGBoost model after PCA dimensionality reduction is more in line with the original value curve and can also fit the original data in the prediction of the temperature peaks of the 60th, 200th, and 350th time segments. Although the changing trend of the XGBoost model can be consistent with the original curve, its prediction curve is still relatively deviated from the original curve. The PSO-XGBoost model has a certain error with the original temperature data in the peak prediction of the 200th and 350th time segments, and the effect is not as good as that of PCA-PSO-XGBoost.

Table 6 shows that on the high-temperature dataset, the PCA-PSO-XGBoost temperature prediction model proposed in this paper outperforms the XGBoost and PSO-XGBoost prediction models on every metric. The prediction accuracy RMSE, MSE, *R*^2^, and MAE of PSO-XGBoost are 0.1676, 0.0281, 0.9969 and 0.1495, respectively, which are 87%, 98%, 20%, and 88% higher than XGBoost. This shows that on higher temperature datasets, the search optimization of the XGBoost parameters by PSO can also significantly improve the model accuracy. The prediction accuracy RMSE, MSE, *R*^2^, and MAE of PCA-PSO-XGBoost are 0.0104, 0.0001, 0.9999, and 0.0036, respectively, and its RMSE, MSE and MAE values are 59%, 99%, and 98% higher than those of PSO-XGBoost, which proves that on the higher temperature data set, after the PCA dimensionality reduction process, the influence of redundant variables is eliminated, which can effectively improve the model accuracy of PSO-XGBoost. These verify that the PCA-PSO-XGBoost model proposed in this paper can also have good accuracy in higher temperature prediction.

## 4. Conclusions

The temperature is time-varying, lag, strong interference, and multi-coupling. When the measured temperature changes suddenly, the output of the thermal resistance in the temperature sensor will be delayed for a period of time, which causes the temperature data to have a hysteresis. The commonly used linear model cannot effectively predict the dynamic change law of the sheep house environment. This paper selects XGBoost to predict the temperature change to solve this problem. It uses the PCA dimensionality reduction algorithm to screen the sheep house environmental parameters. Temperature affects key environmental factors. The PSO optimization technique is used to perform a worldwide search for XGBoost parameters to forecast and assess the temperature of a sheep barn in Xinjiang, avoiding the empirical and randomness of XGBoost model parameter selection. Comparing a single SVR, PSO-BPNN, PSO-SVR, and PSO-XGBoost, the experimental findings demonstrate that:

(1) By using the PCA dimensionality reduction algorithm, the key environmental factors affecting the temperature of the sheep house can be screened out as air temperature, air humidity, carbon dioxide concentration, PM10, PM2.5, and the total suspended particulate matter (TSP), which can eliminate the correlation between the environmental factors. When inputting model parameters, irrelevant factors such as light intensity, noise, and H_2_S can be filtered, which can reduce the number of input variables, reduce the complexity of the model, simplify the model structure, and avoid the dimensional disaster caused by excessive feature dimensions. All of these benefits are crucial for increasing the model’s accuracy. At the same time, this also means that the use of light intensity sensors, noise sensors, and H_2_S sensors can be reduced, and the cost of sensor placement and energy consumption can be saved.
(2) The global search for the parameters of the XGBoost model using the PSO optimization algorithm can significantly increase model accuracy compared to the training results of a single XGBoost model. Avoid the empirical and randomness of parameter selection and eliminate the correlation between the predicted temperature value and the actual value. The developed PSO-XGBoost model has excellent prediction effectiveness, strong stability, and practical applicability.
(3) BPNN and SVR need to configure multiple parameter items and have many training times to improve prediction. Simultaneously, the XGBoost parameter setting is relatively simple, and the fitting effect is good; the model has high precision. Compared with PSO-BPNN and PSO-SVR, the evaluation indicators RMSE, MSE, *R*^2^, and MAE of PSO-XGBoost are 0.1256, 0.0158, 0.9957, and 0.0898, respectively. The model accuracy is higher, indicating that it has more performance advantages and practical application value.
(4) In essence, BPNN is a gradient descent algorithm. The optimized objective function is complicated, the convergence speed is sluggish, and it is possible to fall into local extreme values, leading to the failure of network training. The training effect of the SVR model is overly dependent on parameter selection, and the variation in parameter selection directly impacts the model’s predictive performance and generalizability. Based on conventional Boosting, XGBoost employs CPU multi-threading and provides a regularization term that regulates the model’s complexity and prevents over-fitting. Therefore, it can effectively improve the accuracy of the model. In small-sample prediction, a single XGBoost can better mine the hidden information between the data than a single BPNN and a single SVR and has a better fit.

The sheep house temperature prediction model based on PCA-PSO-XGBoost proposed in this paper can practically provide guiding suggestions for monitoring and regulating temperature changes in intensive housing and can be extended to the prediction research of other environmental parameters of other animal houses such as pig houses and cow houses in the future.

## Figures and Tables

**Table 1:** Part of experimental original data collected on 8–22 February 2021.

Time	Air Temperature	Air Humidity	CO_2_	PM_2.5_	PM_10_	Light Intensity	Noise	TSP	H_2_S
8 February 2021 17:11:56	4.2	78	1355	16.3	81.3	146	31.1	120.7	5.2
8 February 2021 17:21:35	3.9	78.4	1330	26.5	107.1	97	72.7	165.3	5.2
-	-	-	-	-	-	-	-	-	-
22 February 2021 17:12:15	6.6	81.2	855	12.5	24	97	40.2	45.3	5.2
22 February 2021 17:22:15	6.4	81.4	780	9.3	24.6	183	34.1	42	5
22 February 202117:32:16	6.3	81.4	770	12.2	34.8	159	31.8	58.2	1.4

**Table 2:** PCA contribution rates and Eigen values.

Component	Initial Eigenvalues			Extract the Load Sum of Squares			Rotational Load Sum of Squares		
	Total	Variance/%	Accumulation/%	Total	Variance/%	Accumulation/%	Total	Variance/%	Accumulation/%
1	3.151	35.016	35.016	3.151	35.016	35.016	2.938	32.650	32.650
2	2.164	24.043	59.060	2.164	24.043	59.060	2.310	25.666	58.315
3	1.605	17.832	76.892	1.605	17.832	76.892	1.672	18.576	76.892
4	0.952	10.579	87.470	-	-	-	-	-	-
5	0.491	5.460	92.930	-	-	-	-	-	-
6	0.391	4.342	97.272	-	-	-	-	-	-
7	0.187	2.076	99.348	-	-	-	-	-	-
8	0.059	0.652	100.000	-	-	-	-	-	-
9	0.000	0.000	100.000	-	-	-	-	-	-

**Table 3:** Component matrix.

Parameter	Component		
	1	2	3
TSP	0.995	−0.077	0.018
PM_10_	0.988	−0.078	−0.006
PM_2.5_	0.972	−0.068	0.103
CO_2_	0.030	0.901	0.188
Air humidity	−0.141	0.864	−0.179
Light intensity	0.031	−0.799	0.131
Noise	0.039	−0.299	0.013
Air temperature	0.016	−0.047	0.904
H_2_S	−0.062	0.062	−0.871

**Table 4:** Correlation matrix.

Correlation Matrix										
		Air Temperature	Air Humidity	CO_2_	PM_2.5_	PM_10_	Light Intensity	Noise	TSP	H_2_S
Correlation	Air temperature	1.000	−0.211	0.141	0.122	0.011	0.168	0.025	0.035	−0.597
	Air humidity	−0.211	1.000	0.715	−0.209	−0.200	−0.575	−0.157	−0.204	0.189
	CO_2_	0.141	0.715	1.000	−0.019	−0.044	−0.574	−0.189	−0.039	−0.040
	PM_2.5_	0.122	−0.209	−0.019	1.000	0.940	0.107	0.035	0.963	−0.139
	PM_10_	0.011	−0.200	−0.044	0.940	1.000	0.094	0.068	0.997	−0.068
	Light intensity	0.168	−0.575	−0.574	0.107	0.094	1.000	0.108	0.098	−0.106
	Noise	0.025	−0.157	−0.189	0.035	0.068	0.108	1.000	0.061	−0.049
	TSP	0.035	−0.204	−0.039	0.963	0.997	0.098	0.061	1.000	−0.085
	H_2_S	−0.597	0.189	−0.040	−0.139	−0.068	−0.106	−0.049	−0.085	1.000

**Table 5:** Experimental results for different prediction models.

Predict Model	Error Type			
	RMSE	MSE	*R* ^2^	MAE
BPNN	1.2354	1.5261	0.5845	0.9449
SVR	1.1422	1.3046	0.6448	0.5718
XGBoost	0.1373	0.0189	0.9949	0.1034
PSO-SVR	0.7035	0.4950	0.8652	0.2454
PSO-BPNN	0.4470	0.1998	0.9456	0.2344
PSO-XGBoost	0.1256	0.0158	0.9957	0.0898
PCA-PSO-XGBoost	0.0433	0.0019	0.9995	0.0065

**Table 6:** Experimental results for different prediction models in high temperature dataset.

Predict Model	Error Type			
	RMSE	MSE	*R* ^2^	MAE
XGBoost	1.2562	1.5782	0.8312	1.2088
PSO-XGBoost	0.1676	0.0281	0.9969	0.1495
PCA-PSO-XGBoost	0.0104	0.0001	0.9999	0.0036

## Data Availability

The data presented in this study are available on request from the corresponding author. The data are not publicly available due to company policy.
